# Supplementary material for: WordCommentsAnalyzer: A windows software tool for qualitative research
Source: F1000Res. 2018 Sep 4;7:536. Originally published 2018 May 3. [Version 2] doi: 10.12688/f1000research.14819.2 (PMC6137411; doi:10.12688/f1000research.14819.2)
Supplement: Supplementary file 3 [file f1000research-7-17614-s0002.tgz › b2a79496-4ada-47c4-9682-a99ce867836d.zip!/Eight Iranian Health Journals/Archives of Rehabilitation_69.docx]

© Archives of Rehabilitation. This work is licensed under CC BY-NC 4.0 (https://creativecommons.org/licenses/by-nc/4.0/).

Volume & Issue: دوره 2 - شماره 3 و 4

Title: بررسی کودک‌آزاری جسمانی در مراجعان سه درمانگاه تخصصی اورژانس کودکان در شهرستان تهران

http://rehabilitationj.uswr.ac.ir/article-1-543-fa.html

هدف: این پژوهش یک مطالعه مقطعی است که به منظور بررسی وضعیت کودک آزاری جسمانی در کودکان شهر تهران، در آذر 1379 به مدت ده روز انجام گرفته است. روش بررسی: در این پژوهش، 45 دستیار آموزش دیده تخصصی کودکان، 3019 کودک زیر 18سال مراجعه کننده به سه مرکز آموزشی کودکان تهران ( بیمارستان های بهرامی، حضرت علی اصغر (ع) و مرکز طبی کودکان ) را مورد مطالعه قرار دادند که از این تعداد، 1578 پسر و 1441 دختر بودند. با انجام مصاحبه بالینی، معاینه فیزیکی و تکمیل پرسش نامه بررسی کودک آزاری جسمانی روی مراجعان این مراکز، در مجموع 367 کودک، یعنی 12/2 درصد آنها ( 14/5درصد پسران و 9/6 درصد دختران) دچار آزار جسمانی شده بودن. یافته‌ها: علائم آسیب، بیشتر در صورت، اندام فوقانی، پشت و اندام تحتانی بود. شدت آسیب وارده، 12/8درصد موارد شدید و 87/2 درصد موارد در حد متوسط بوده است. والدین بیش از سایرین، عامل کودک آزاری جسمانی در خانواده کودکان آسیب دیده، بیشتر مربوط به پر اولاد بودن، اعتیاد یکی از اعضای خانواده و وجود استرس حاد و شدید در طول 6ماه گذشته بوده است. نتیجه‌گیری: با انجام آزمون 2x ارتباط معنی داری بین اقدام به کودک آزاری جسمانی باسن، جنس، محل سکونت، جدایی والدین و سابقه آزار جسمانی در کودکی والدین، مشاهده شد(001/0>pvalue)

Title: سنجش میزان رضایت خدمت گیرندگان مجتمع های توانبخشی روزانه شهر تهران با دیدگاه مشتری گرایی

http://rehabilitationj.uswr.ac.ir/article-1-545-fa.html

هدف: پژوهش حاضر در راستای دیدگاه مشتری‌گرایی به اجرا درآمده است و هدف آن سنجش میزان رضایت خدمت‌گیرندگان 12 مجتمع توانبخشی روزانه شهر تهران به منظور بهبود فرآیندهای ارائه خدمات بر اساس نظر خدمت‌گیرندگان است که در نهایت، می‌تواند زمینه مناسبی برای دخالت دادن آنان در فرآیند ارائه خدمات فراهم کند. روش بررسی: جامعه نمونه، 133 نفر از خدمت گیرندگان بودند که با روش نمونه‌گیری تصادفی با طبقه‌بندی انتخاب شدند. چون قبلا مطالعه‌ای در این زمینه انجام نگرفته بود، یک پرسشنامه نگرش‌سنج با مقیاس 5 درجه لیکرت شامل 92 سوال و یک سوال باز پاسخ، تهیه و در یک بررسی مقدماتی، میانگین نمره رضایت (3/71) و انحراف واریانس آن (0/297) تعیین شد. ضمنا پس از حذف سوال‌های فاقد همبستگی، تعداد آنها به 68 سوال کاهش یافت. یافته‌ها: تجزیه و تحلیل اطلاعات با استفاده از نرم‌افزارهای Excell/ win SPss 7.5 win، میزان بالای رضایت را در بسیاری حوزه‌ها نشان داد، اما، موارد عدم رضایت، یا نقص را نیز مشخص کرد. نتیجه‌گیری:‌ متغیرهای سن، جنس و تحصیلات تاثیر تعیین‌کننده‌ای بر سطوح رضایت نداشتند. سوال باز نیز تعدادی از حوزه‌های عدم رضایت را مشخص کرد و خدمت‌گیرندگان، پیشنهادهایی برای اصلاح و بهبود فرآیندها ارائه دادند.

Title: گزارش غربالگری شیر خواران ( ۱۸ – ۴ ماهه) شهر کرج از نظر تأخیر تکامل حرکتی

http://rehabilitationj.uswr.ac.ir/article-1-546-fa.html

این مطالعه حاصل بررسی فرآیندها به منظور غربالگری شیرخواران 18-4 ماهه شهر بزرگ کرج از نظر تاخیر تکامل حرکتی در سال 1379 می‌باشد. هدف از این مطالعه، غربالگری شیرخواران به منظور تشخیص زودرس و در نتیجه، درمان توانبخشی به موقع، قبل از تثبیت ضایعات، محاسبه اعتبار و تکرارپذیری و قابلیت انجام آزمون INFANIB در ایران بوده است. در این مطالعه که با همکاری شبکه  بهداشت و درمان و اداری بهزستی شهرستان کرج انجام شد، برای معرفی شیرخواران به پایگاه های طرح، از همکاری درمانگاه ها پایگاه های بهداشتی شبکه استفاده شد و بدین ترتیب، تمامی شیرخواران 18-4 ماهه که در طی ماه های خرداد تا دی سال 1379 برای دریافت مراقبت های بهداشتی به مراکز بهداشت شبکه مراجعه کردند، مورد بررسی قرار گرفته اند. این شیرخواران مجموعا 6150 نفر (معادل نصف جمعیت گروه هدف) بودند که از نظر میزان تکامل حرکتی توسط کاردرمانگران با استفاده از INFANIB SCONING بررسی شدند. مطالعه حاکی از آن است که میزان شیوع تاخیر تکامل حرکتی در شهرستان کرج 8/1000 است که به طور معنی داری بالاتر از آمار جهانی است. هم چنین، انجام آزمون INFANIB در ایران در مقایسه با آزمون های استاندارد دارای 90% حساسیت و 81% اختصاصی بودن و 85% ارزش اخباری مثبت و 87% ارزش اخباری منفی و در نتیجه معتبر است. هم چنین، بر اساس این مطالعه، نتایج معاینات متخصص کودکان و کاردمانگر یکسان است و در نتیجه، تست تکرارپذیر نیز هست. متوسط زمان لازم برای انجام معاینه و نمره گذاری به این روش، حدود 6-5 دقیقه است. بنابراین INFANIB Sconing به دلیل کمی بودن، صرف زمان کوتاه و توانایی کاردرمانگر، فیزیوتراپ، پرستار و پزشک در انجام آن، همچنین حساسیت اختصاصی بودن بالا، به عنوان روش مطلوب غربالگری تاخیر تکامل حرکتی در مراکز بهداشتی – درمانی پیشنهاد می شود.

Title: بررسی میزان شیوع افسردگی وعوامل مؤثر بر آن در دانش آموزان پسر مقطع پیش دانشگاهی شهرستان کازرون

http://rehabilitationj.uswr.ac.ir/article-1-547-fa.html

هدف: پژوهش حاضر، یک مطالعه توصیفی، تحلیلی و مقطعی است که در بهمن ماه 1379 انجام گرفته است. هدف از این پژوهش، تعیین میزان شیوع افسردگی و عوامل موثر بر آن در دانش آموزان پسر مقطع پیش دانشگاهی شهرستان کازرون است. روش بررسی: به منظور دستیابی به هدف فوق 240 دانش آموز پسر مقطع پیش دانشگاهی از طریق نمونه گیری طبقه ای تصادفی انتخاب و توسط پرسشنامه دموگرافیک و مقیاس افسردگی زونگ مورد مطالعه قرار گرفتند. یافته‌ها: نتایج این پژوهش نشان داد که 42/9 درصد دانش آموزان جامعه مورد مطالعه داری افسردگی هستند و فاصله اطمینان 95% (49/15%-36/6%) است. شدت افسردگی در جامعه مورد مطالعه به این ترتیب است که %28/8 دارای افسردگی عمیق بوده اند، استفاده از آزمون 2x نشان داد که افسردگی در دانش آموزان مورد مطالعه با وضعیت مالی خانواده، چگونگی رابطه پدر و مادر، مطرح کردن مشکلات با دیگران، میزان نگرانی در مورد رفتن به خدمت سربازی، میزان نگرانی در مورد موفقیت در کنکور، نحوه برخورد دبیران و مسئولان مدرسه، و میزان تحصیلات مادر ارتباط معنی داری دارد. نتیجه‌گیری: سپس با ورود این متغیرها در یک مدل رگرسیون لجستیک این نتیجه به دست آمد که چهار متغیر وضعیت مالی خانواده، چگونگی رابطه پدر و مادر، مطرح کردن مشکلات با دیگران و میزان نگرانی در مورد رفتن به خدمت سربازی دارای اثر فرآینده، روی ابتلا به افسردگی هستند. ظرفیت مدل فوق این است که نه تنها متغیرهای تاثیر گذار بر ایجاد افسردگی مورد شناسایی قرار گرفته اند، بلکه با داشتن اطلاع از وضعیت دانش آموز می توان احتمال ابتلا به افسردگی را با استفاده از این الگو، تخمین زد.

Title: بررسی میزان اثر تحریک ارتعاشی و تحریک الکتریکی بر روی حداکثر انقباض ارادی

http://rehabilitationj.uswr.ac.ir/article-1-548-fa.html

هدف: میزان تغییر حداکثر انقباض ارادی به دنبال اعمال تحریکات ارتعاشی عضله آگونیست و تحریک الکتریکی عضله مقابل به طور جداگانه و کاربرد توام آنها به طور هم زمان، مورد تحقیق و بررسی قرار گرفت. روش بررسی: در این تحقیق 45 نفر از افراد سالم مورد بررسی قرار گرفتندکه به سه گروه 15 نفری تقسیم شدند. در هر گروه، قبل از انجام آزمایش، از فرد خواسته شد که حداکثر انقباض ارادی را در عضله گاستروکنمیوس داشته باشد که به صورت سیگنال‌های EMG ثبت شد. یافته‌ها: در گروه اول، تاندون آشیل به مدت 15 دقیقه مورد ارتعاش (100 هرتز و دامنه 0/8 میلی‌متر) قرار گرفت. مشخص شد که MVC عضله گاستر کنمیوس در اثر ارتعاش کاهش یافته، اما بعد از 15 دقیقه تحریک الکتریکی عصب پرونئال در گروه 2، تغییری نکرده است. در گروه سوم، در اثر کاربرد توام دو مداخله، MVC این عضله نیز تغییری نکرد. نتیجه‌گیری: به طور کلی، اعمال ارتعاش بر عضله، در مقایسه با دو روش دیگر، سبب کاهش بیشتری در MVC می‌شود.

Title: بررسی عوامل ایجادکننده شکستگی‌های هیپ در سالمندان بستری در مراکز آموزشی درمانی شهرستان‌های کرمان و رفسنجان در سال‌های 79 تا 80

http://rehabilitationj.uswr.ac.ir/article-1-549-fa.html

هدف: شکستگی هیپ، شایع ترین علت مرگ ناشی از ضربه، بعد از 75 سالگی است که اغلب در زنان مشاهده می شود و می تواند عوارض منفی عمیقی روی نحوه زندگی آنان باقی بگذارد. هدف از این مطالعه، بررسی عوامل ایجاد کننده شکستگی های هیپ در سالمندان بستری در بخش های ارتوپدی مراکز آموزشی و درمانی شهرستان کرمان و رفسنجان بود. روش بررسی: برای انجام مطالعه از روش توصیفی استفاده شد. برای تمام بیماران مبتلا به شکستگی های هیپ (257نفر) که در طی 27ماه، (از اول اردیبهشت سال 77تا پایان تیرماه 79) در بخش های ارتوپدی مراکز آموزشی و درمانی حضرت علی ابن ابیطالب (ع) رفسنجان و شهید باهنر کرمان بستری شده بودن، پرسش نامه ای شامل 20سوال (درمورد عوامل ایجاد کننده شکستگی هیپ) تکمیل شد. این عوامل عبارت بودند از : سن، جنس، نوع شکستگی، پوکی استخوان، میزان مصرف شیر، آتروفی عضلانی، خطرات محیط زندگی، شاخص توده بدنی، بیماری های اعصاب، دیابت، سابقه شکستگی، مصرف سیگار، داروهای ضد تشنج و ضد افسردگی، بیماری قلبی، تحرک و فعالیت کم، سابقه معلولیت، ضعف ادراک سن یائسگی، اختلالات بینایی و استفاده از محاقظ خارجی هیپ. یافته ها: نتایج این مطالعه نشان می دهد که تعدادی از عوامل فوق از نظر تاثیر بر در شکستگی هیپ سالمندان، از درصد بالاتر(بیشتر از 50درصد) برخوردار هستند. این عوامل شامل، خطرات محیط زندگی (%81/7)، آتروفی عضلانی (%72/8)، شکستگی های قبلی (%52/1)، تحرک و فعالیت کم (%70/8)، مصرف کم شیر (%100)، شاخص توده بدنی پایین(%79/8)، پوکی استخوان (%52/9) و عدم استفاده از محافظ خارجی (%100) است. نتیجه گیری: یافته های این مطالعه پیشنهاد می کند که محیط زندگی سالمندان باید اصلاح شود. به عنوان مثال، محیط زندگی آنان باید روشنایی کافی داشته باشد تا از سقوط آنها پیشگیری شود. نرده های ودستگیره های محکم در مسیر رفت و آمد آنان وجود داشته باشد تا مانع سقوط آنها شود. به از ورزش و جریان الکتریسته نوع فارادیک برای جلوگیری از آتروفی عضلانی و بهبود آمادگی بدنی، قدرت عضلانی، تعادل و هم آهنگی آنان می توان استفاده کرد. در مان اختلال بینایی مهم است، زیرا خطر سقوط را کاهش می دهد. ورزش های تحمل وزن مانند، راه رفتن، مصرف کافی شیر، دریافت کلسیم، پوکی استخوان جلوگیری می کند و متعاقبا شکستگی هیپ را کاهش می دهد. استفاده از محافظ خارجی هیپ (پد) در سالمندان بسیار اهمیت دارد، زرای انرژی ناشی از سقوط را جذب می کند و در نتیجه، می تواند شکستگی هیپ را کاهش دهد.

Title: بررسی آگاهی بیماران مبتلا به صرع از بیماری خود در مراجعان کلینیک صرع بیمارستان رازی تبریز

http://rehabilitationj.uswr.ac.ir/article-1-550-fa.html

اهداف: میزان آگاهی بیماران مبتلا به صرع از بیماری خود، یک اصل ضروری در درمان آنهاست. مطالعات انجام گرفته در این زمینه، بسیار اندک هستند. در این جا اولین مطالعه در کلینیک صرع بیمارستان رازی تبریز راجع به میزان آگاهی بیماران ایرانی مبتلا به صرع از بیماری خود گزارش می شود. روش یررسی: برای انجام مطالعه، 750 بیمار با سن بالای 15 انتخاب شدند. روش جمع آوری داده ها، پرسش نامه استاندارد شده (Epilepsy patients knowledge questionnaire) دانشگاه اوهایو بود که حاوی اجزای دموگرافیک و آگاهی بیماران از تعرریف تشنج، نوع و مقدار داروهای مصرفی، نحوه برخورد با عوارض داروها و عوامل ازاینده و یا کاهنده تشنج و جوانب قانونی رانندگی و استخدامی مبتلایان به صرع بود. پس از جمع آوری اطلاعات که به طریق مصاحبه با بیماران و درج اطلاعات انجام گرفت، محاسبات آماری با استفاده از نرم افزار 16ep برای بررسی میزان آگاهی بیماران و رابطه آن با سن، جنس، میزان تحصیلات و سال های ابتلا به صرع، مورد ارزیابی قرار گرفت. یافته ها: اهم نتایج حاصله عبارت اند از: در 750بیمار مورد بررسی (280  مورد مونث و 470 مورد مذکر) سن و تعداد متوسط سال های با صرع ، به ترتیب 12=10,32=10 سال بود. تعدا سال های با صرع و سن بیماران، در میزان آگاهی آنها تاثیری نداشت ( 2/0=  r , 01 .0 >p )میزان تحصیلات ( 2/0=  r , 01 .0 >p ) و جنسیت 01 .0 >p آگاهی بیماران مذکر در مقایسه بیشتر بود عوامل موثری در آگاهی بیماران بودن. بیست درصد افراد، صرع درا یک اختلال مغزی می دانستند. سی و هشت درصد افراد می دانستند که عوامل افزایند تشنج چه مواردی هستند. پایین ترین نمرات مربوط به سوال های طرز برخورد با عوارض دارویی و زمان مناسب قطع داروهای ضد تشنج بود. (فقط5  در صد افراد آگاهی کافی داشتند). چهل درصد بیماران از خطرات رانندگی افراد مصورع آگاه بودن به طور کلی، سطح آگاهی بیماران پایین بود. نتیجه گیری: بیماران مبتلا به صرع از بیماری خود آگاهی کافی ندارند و این مسئله روی پذیرش آنها برای دریافت درمان مناسب، کاهش آسیب های ضمن بروز حمله و درکل، روی پیشگیری در درمان حملات تشنجی تاثیر بسزایی دارد. بنابراین، افزایش سطح آگاهی آنها یک ضرورت است، به خصوص لازم است در مورد این که چگونه از آسیب های وارده ضمن حمله جلوگیری کنند، با عوارض دارویی چگونه برخورد کنند و تا چه زمانی باید درمان ضد تشنج را ادامه دهند، هم چنین راجع به ملاحظات قانونی رانندگی و اشتغال به حرف مختلف، آگاهی کافی به دست آورند.

Title: تغییرات توجه و حافظه در اوتیسم

http://rehabilitationj.uswr.ac.ir/article-1-551-fa.html

مطالعه حاضر مروری است و آخرین یافته های مربوط به تغییرات توجه و حافظه در بیماران مبتلا به اوتیسم که امروز منتشر شده اند. مهم ترین تغییرات توجه در اوتیسم : اختلال عملکرد در سیستم تنظیم کننده برانگیختگی در حالات بیشتر و کمتر از حد معمول کاهش تطبیق یافتن و پردازش محرکات جدید توجه بیش از حد به بعضی از محرکات محیطی کم ارزش، به علت عدم درک معنای محرکات مشخص اجتماعی مهمترین تغییرات حافظه در اوتیسم : حافظه عادتی بهنجار نقص در حافظه کارآمد نقص در حافظه ضمنی و صریح یادآوری ضعیف تر مطالب در وضعیت یادآوری آزاد نسبت به یادآوری با سرنخ اشکال در حافظه کلامی در موضوعات سازمان دهی شده از نظر معنایی عملکرد بالای حافظه در بعضی از بیماران در زمینه هایی نظیر، از برخواندن، توانایی خواندن آهنگ یا شناخت قطعات متفاوت موسیقی

Title: تطابق یا سازگاری عصبی

http://rehabilitationj.uswr.ac.ir/article-1-552-fa.html

طبق اصول اختصاصی بودن تمرین specificity و سازگاری‌های اختصاصی به نیازهای تحمیلی (SAID) تطابقات فراوانی در داخل عضله در پاسخ به تمرین های قدرتی رخ داده که سه دسته اصلی آنها شامل: تطابق عصبی، تطابق عضلانی و تطابق متابولیک است. مقدم ترین این سازگاری های ذکر شده، تطابق عصبی است. در این مقاله، ابتدا به بحث در مورد نظریه های مختلفی که تاکنون در زمینه مکانیسم های احتمالی سازگاری عصبی ارائه شده اند، پرداخته می شود و ارتباط زمانی تطابق عصبی و عضلانی و عوامل موثر بر آنها در اشخاص سالم و بیمار، به تفصیل مورد بررسی قرار می گیرد. تشریح اصول حاکم بر تمرین های قدرتی و مضرات این تمرین ها از دیدگاه فیزیوتراپی، آخرین دست آورد این مقاله خواهد بود.

Title: بررسی ۲۰ مورد مولتیپل اسکلروزیس کودکان در بیمارستان مفید تهران

http://rehabilitationj.uswr.ac.ir/article-1-553-fa.html

هدف: یک نوع بیماری نادر در کودکان زیر 10 سال است و غالبا در سنین بعد از قاعدگی و در دهه سوم گزارش شده است. این بیماری روی میلین سیستم عصبی مرکزی اثرگذار است و علت پاتوژنز آن نامعلوم است. شاید علل اتوایمیون و یا ویرال در ایجاد بیماری موثر باشد. ضایعات مولتیپل اسکلروزیس در زمان و مکان توسعه می یابند و شدت تظاهرات بالینی متفاوت دارند و از یک ناخوشی جزئی تا یک بیماری پیشرونده ناتوان کننده متغیرند. روش بررسی: در این مطالعه گذشته نگر توصیفی – مقطعی، 20مورد از بیماری مولتیپل اسکلروزیس (MS) در سال های 1376 تا 1379 در بخش فوق تخصصی اعصاب کودکان بیمارستان مفید مورد پیگیری قرار گرفته است (14دختر و 6پسر) در این مطالعه، پایین ترین تظاهرات سنی، 4 سالگی و بالاترین، 13 سالگی بوده است. یافته‌ها: برای کلیه بیماران MRIمغز انجام شده است و وجود پلاک های متعدد مغزی، مبین تشخیص بوده است. ضمنا برای همه بیماران (V.E.R) انجام گرفت و افزایش Latency در اکثر بیماران گزارش شده است. شایع ترین علائم بالین عبارتند از: افزایش رفلکس عمقی و تری تاندونی، آتاکسی، دوبینی، کاهش قدرت عضلات، اختلال راه رفتن، سرگیجه و پارستزی. برای کلیه بیماران، CBC، الکترولیت های سرم ، Anti Lecell  .ANA.Dnase و بررسی مایع مغزی – نخاعی در خواست شده است و بر اساس ضرورت در برخی بیماران (S.S.E.P) و (A.B.R) و Spinal  MRI نیز انجام گرفته است. نتیجه‌گیری: در این مطالعه، سعی شد که این بیماری نادر بر اساس علائم کلینیکی و الکترودیاگنوزیس و Neuro Imaging نوصیف شود تا متخصصان امر در برخورد با این علائم به فکر M.S حتی در کودکان نیز باشند.
